# Genetic variation in the pleiotropic association between physical activity and body weight in mice

**DOI:** 10.1186/1297-9686-41-41

**Published:** 2009-09-23

**Authors:** Larry J Leamy, Daniel Pomp, J Timothy Lightfoot

**Affiliations:** 1 Department of Biology, University of North Carolina at Charlotte, Charlotte, North Carolina 28223, USA; 2 Department of Genetics, University of North Carolina, Chapel Hill, NC 27599, USA; 3 Department of Nutrition, University of North Carolina, Chapel Hill, NC 27599, USA; 4 Department of Cell and Molecular Physiology, University of North Carolina, Chapel Hill, NC 27599, USA; 5 Carolina Center for Genome Science, University of North Carolina, Chapel Hill, NC 27599, USA; 6 Department of Kinesiology, University of North Carolina at Charlotte, Charlotte, North Carolina 28223, USA

## Abstract

**Background:**

A sedentary lifestyle is often assumed to lead to increases in body weight and potentially obesity and related diseases but in fact little is known about the genetic association between physical activity and body weight. We tested for such an association between body weight and the distance, duration, and speed voluntarily run by 310 mice from the F_2 _generation produced from an intercross of two inbred lines that differed dramatically in their physical activity levels.

**Methods:**

We used a conventional interval mapping approach with SNP markers to search for QTLs that affected both body weight and activity traits. We also conducted a genome scan to search for relationship QTLs (*rel*QTLs), or chromosomal regions that affected an activity trait variably depending on the phenotypic value of body weight.

**Results:**

We uncovered seven quantitative trait loci (QTLs) affecting body weight, but only one co-localized with another QTL previously found for activity traits. We discovered 19 *rel*QTLs that provided evidence for a genetic (pleiotropic) association of physical activity and body weight. The three genotypes at each of these loci typically exhibited a combination of negative, zero, and positive regressions of the activity traits on body weight, the net effect of which was to produce overall independence of body weight from physical activity. We also demonstrated that the *rel*QTLs produced these varying associations through differential epistatic interactions with a number of other epistatic QTLs throughout the genome.

**Conclusion:**

It was concluded that individuals with specific combinations of genotypes at the *rel*QTLs and *epi*QTLs might account for some of the variation typically seen in plots of the association of physical activity with body weight.

## Background

Mounting evidence suggests that physical activity is crucial for the health and well being of people of all ages, from very young children [1] to elderly adults [2]. Physical inactivity is well known to be associated with a diverse number of health problems such as coronary heart disease and colon cancer [3-6] and has been ranked as the second leading actual cause of death in the United States [7]. Sedentary lifestyles also are thought to promote obesity and associated diseases such as diabetes that have become a special concern in recent years because of their dramatic increase in frequency even in children [8]. Moreover, some studies have demonstrated beneficial effects of physical activity independent of body weight or weight gain [9]. Given the medical ramifications of obesity, therefore, it is clearly important that we have a better understanding of the association between physical activity and weight.

The genetic contribution to the physical activity/body weight relationship is of particular interest, especially because it may account for some of the variability in weight typically observed among individuals with increased levels of physical activity. The question is, do genes or gene interactions with pleiotropic effects on both physical activity and weight traits exist or do these two traits have completely separate genetic bases? At present, we have little information to answer this question and in fact only in recent years has the genetic basis of physical activity itself been seriously explored. To date, various genetic association studies have led to the identification of more than 30 potential candidate genes in humans influencing physical activity traits such as endurance and speed [8]. However, some of these data are equivocal and it remains to be seen whether the effects of many of these genes on physical activity traits will be verified in subsequent studies and/or whether they also influence body weight.

Lightfoot *et al*. [10] have taken an alternative approach to explore the genetic basis of physical activity by conducting a quantitative trait locus (QTL) study in mice. Using an F_2 _population generated from an original cross of two inbred strains differing dramatically in their physical activity levels, these investigators uncovered several different QTLs controlling the distance, duration, and speed voluntarily run by the mice. Most recently, Leamy *et al*. [11] followed up a QTL analysis with a full-genome scan for epistasis in this same population of mice and discovered a number of epistatic interactions of unknown QTLs that significantly affected the physical activity traits. Contribution of epistasis to the total variation of the traits (average of 26%) was about the same as that for single-locus effects of QTLs, suggesting that epistatic interactions of genes may be an important component of the genetic basis of physical activity [11]. Although not genetically analyzed, body weights were also recorded for all the F_2 _mice, and thus this population presented a unique opportunity to investigate the genetic association between the physical activity traits and weight.

**Figure 1:**
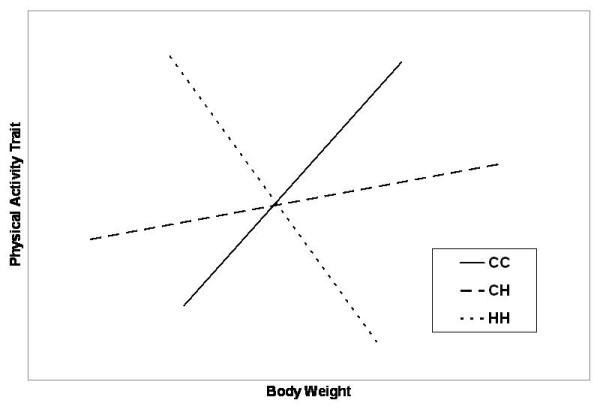
**A hypothetical example of the variation in the effects of the genotypes at a relationship QTL on the association between physical activity and body weight**. HH and CC = homozygotes, CH = heterozygotes; note that the effects of different values of body weight are opposing and cancel each other.

We conducted such an investigation in several steps, the first of which was to map direct-effect QTLs for body weight in these mice to determine whether any were at the same location as those affecting the physical activity traits (suggesting common QTLs with pleiotropic effects). A second step was to conduct a genome search for relationship QTLs or *rel*QTLs [12,13], regions in the genome that affect the physical activity traits variably depending on the phenotypic value for body weight. The effects of a *rel*QTL may be visualized by regressions of the dependent variable (physical activity trait) on an independent variable (body weight) that differ for each of several genotypes. For illustrative purposes, Figure 1 depicts a hypothetical situation where the relationship between a physical activity trait and body weight is positive for one homozygote (designated CC) but negative for the other homozygote (HH) at a *rel*QTL locus. Basically *rel*QTLs produce their effects by interacting with other genes (differential epistasis) or with the environment [14,12]. Since epistatic interactions of QTLs were previously found to affect the physical activity traits in these mice [11], it seemed reasonable to test for differential epistatic effects as a potential explanation for any *rel*QTLs discovered [13]. Thus, as a third and final step, we screened the genome to see if *rel*QTLs interacted with other epistatic QTLs (*epi*QTLs) to significantly affect the physical activity traits or body weight.

## Methods

### The population and traits

The F_2 _population of mice used in this study was generated from crossing two inbred strains, C57BL/J and C3H/HeJ, previously identified as exhibiting considerable divergence in measures of physical activity. Reciprocal crossing of mice from these strains resulted in 63 F_1 _mice that in turn were crossed to produce a total of 310 F_2 _offspring (all first litters except for four matings that produced two successive litters). All mice were maintained in the University of North Carolina at Charlotte Vivarium at 18-21°C and 20-40% humidity with 12 h light/dark cycles and with food (Harland Teklad 8604 Rodent Diet, Madison, WI) and water provided *ad libitum*.

We measured three physical activity traits in all F_2 _mice during a 21-day interval starting at an average age of 63 days (9 weeks). These traits included total daily distance (kilometers) and total daily exercise time (minutes) that were recorded every 24 h, and average daily running speed (meters/minute) obtained by dividing distance by duration. This was accomplished for all mice with a solid surface running wheel mounted in their cages that interfaced with a computer that counted the total wheel revolutions and recorded the time each mouse spent exercising (see [15] for further details).

Within a week after completion of the phenotyping, the mice were sacrificed, weighed to the nearest 0.1 g, and their kidneys were collected for subsequent DNA extraction. Genotyping of all F_2 _mice was accomplished for 129 single-nucleotide polymorphisms (SNPs) that differed between the C57BL/J and C3H/HeJ *progenitor *strains. These SNPs were chosen to provide a reasonable coverage of the entire genome (including the X chromosome), which they did with an average marker-marker interval of about 14 cM. For all mouse procedures, we followed guidelines approved by the UNC Charlotte Institutional Animal Care and Use Committee and those recommended for ethical use of animals from the American Physiological Society and the American College of Sports Medicine.

### Body weight analyses

As was done previously [10] for the three physical activity traits, we first tested body weight (WT) for potential effects due to sex, litter size, and rearing block. All three factors were entered as classification factors in a linear model and found to be statistically significant. WT was therefore adjusted for the effects of these factors by calculating residuals from the model and adding them to the mean weight in the overall population. This procedure was useful in decreasing non-genetic sources of body size variation and therefore presumably increasing the statistical power to detect QTLs and measure their effects. Merging of the adjusted WT values with the previously adjusted values for the physical activity traits constituted the phenotypic data set used in the analyses described below.

Direct-effect QTL scans for body weight were carried out using the regression approach to interval mapping [16] as previously described for the physical activity traits [10]. Briefly, additive (*X*_*a*_) and dominance (*X*_*d*_) index values first were assigned for C3H/HeJ homozygotes (HH), C57L/J homozygotes (CC), and heterozygotes (CH) at each SNP marker and also imputed for all locations 2 cM apart between flanking markers [10]. Then, we conducted multiple regression of body weight on these index values at each location to test for QTLs, and if present, estimated their effects by calculation of the additive (*a*) and dominance (*d*) genotypic values. The *a *values estimate one-half of the difference between the mean body weights of the two homozygotes and the *d *values estimate the difference between the mean weight of the heterozygotes and that of the mean of the two homozygotes [17]. The model was as follows:(1)

where μ is a constant, *e *= the residual, and the other terms are as defined above.

To test for overall significance at each location, the probabilities generated from the regression analyses were logarithmically transformed to calculate LPR values [(log_10_(1/Prob.)] similar to LOD scores [18]. The highest LPR score on each chromosome was considered to indicate a putative QTL if this score exceeded a specific threshold value (see below). Confidence intervals for each QTL were determined by the one-LOD rule [19]. Each chromosome was also tested for two-QTL and sex-specific QTL effects affecting weight in the manner already described [10].

We used the traditional permutation method of Churchill and Doerge [20] with 1000 shuffles to generate specific 5% threshold values for each chromosome that were suggestive of linkage as well as a 5% genome-wise threshold value that offered significant evidence of linkage. The chromosome-wise values were particularly useful in adjusting for the different sampling of each of the chromosomes that varied in length and density of SNP markers. Further, there is only a 5% chance of a false positive QTL for any LPR score exceeding its chromosome-wise threshold. In addition, given that the chromosomes in our F_2 _population were in linkage equilibrium, only one false positive might be expected over the entire genome of 20 chromosomes. Thus the use of the chromosome-wise threshold values avoids the vast majority of false positive results while suggesting QTL sites that would not be discovered with the use of the much more stringent genome-wide threshold values that basically are designed to eliminate the possibility of false positive results [21,22]. However, as in all QTL studies such as this one, additional studies are invaluable for confirming any putative QTLs identified.

### Relationship QTL scans

To search for relationship QTLs (*rel*QTLs) affecting the association of the physical activity traits with body weight, we used a modification of the regression approach described above. Specifically in these analyses, we regressed the additive and dominance index values, WT, and the *interactions *of body weight with the index values on distance, duration, and speed. Essentially this is an analysis of covariance model where the interest is in the interactions [13]. The model for this approach is represented by the following:(2)

where *y *= the dependent variable and the terms to the right of the operator '|' are partialed out and do not enter into the significance tests and the other terms have been previously defined. Separate analyses were done for the three physical activity traits, and LPR scores were generated as described above and compared to threshold values calculated from permutation procedures run for each trait. Tests for two *rel*QTLs per chromosome as well as sex-specific *rel*QTL effects also were conducted as before.

For those *rel*QTLs affecting two or all three physical activity traits but co-localizing in the same or similar positions, it was useful to conduct pleiotropy tests. We used the procedure outlined by Knott and Haley [23] to test whether separate *rel*QTLs were potentially a common *rel*QTL with pleiotropic effects on several traits. To implement this procedure, we first calculated residual sums of squares from the canonical correlation runs at the most probable location for the individual activity traits to be tested and pooled them into one matrix. We then ran another canonical correlation procedure for the combined traits to obtain a residual sum of squares matrix at the most probable joint location for a *rel*QTL. The pleiotropy test involved a comparison of the determinants of the two matrices with a likelihood-ratio statistic [23]. A significant chi-square value in this test suggested that the QTLs were separate whereas a non-significant value suggested that there could be just one QTL with pleiotropic effects on multiple traits.

For all *rel*QTLs, we were able to quantify genotype-specific associations of the physical activity traits with body weight. This was done by calculating regressions of the physical activity traits on body weight for the HH, CH, and CC genotypes at the SNP loci closest to the locations of all *rel*QTLs. Testing of these regressions was done via individual *t*-tests evaluated at the conventional 5% significance level. The regressions and their associated coefficient of determination (*r*^2^) values were helpful in showing the differences in the associations of the physical activity traits with body weight produced by the three genotypes at each *rel*QTL locus.

### Epistasis scan

One way in which *rel*QTLs can affect the relationship between two traits is by epistatically interacting with other QTLs that differentially affect the traits. This phenomenon is called differential epistasis and has been explained in some detail, including with examples, by Cheverud [24,14]. Therefore, to examine whether differential epistasis might account for the effects of the *rel*QTLs, we scanned the genome for their epistatic interactions with other QTLs (*epi*QTLs) for the trait or traits (including body weight) specifically affected by each of the *rel*QTLs.

The scan was conducted at every location 2 cM apart on all chromosomes (except that of the *rel*QTL) via regression of the trait values on the (fixed) additive and dominance index values for the *rel*QTL (*X*_*ar*_, *X*_*dr*_), the additive and dominance index values at other locations (*X*_*a*_, *X*_*d*_), and the interactions of the two sets of index values. These interactions generated additive by additive (*aa*), additive by dominance (*ad*), dominance by additive (*da*), and dominance by dominance (*dd*) genotypic epistatic components. This model we used was:(3)

where the terms and symbols have already been defined.

Multivariate regression of the combined effects of the four interaction terms generated a Wilk's lambda statistic with its associated probability that was converted to an LPR value used to test for the significance of overall epistasis. Epistasis was considered present when the highest LPR value on a given chromosome exceeded the appropriate (trait- and chromosome-specific) threshold value previously used in testing for *rel*QTLs. If overall epistasis was indicated, we estimated the four individual epistatic components from the regression model and tested them for significance with conventional *t*-tests.

All significant epistatic interactions involving the *rel*QTLs were examined to discover whether they appeared to be acting differentially on the traits. Differential epistasis was assumed to occur for all epistatic interactions affecting only one (activity or weight) trait, but not both traits. In cases where the epistatic interactions were significant for an activity trait and body weight, the direction and magnitude of the four epistatic components for both traits were inspected for potential differences that might indicate differential epistasis. Ideally such comparisons of the epistatic components should be done in a formal statistical test, although past studies have shown that epistatic pleiotropic effects tend to be restricted to single traits [25,13].

## Results

Additional file 1 provides basic statistics for all four traits used in the analyses. On average, the F_2 _mice weighed about 26 grams and ran over 6 km each day during a 330-minute span that generated a speed of 19 meters per minute. As judged by their coefficients of variation (standard deviation/mean, not shown), distance and duration are considerably more variable than speed or body weight. The three correlations between each pair of physical activity traits are positive in sign and moderate to high (especially the 0.92 for distance and duration) in magnitude, and all are statistically significant. However body weight shows no significant association with any of these three activity traits.

### Body weight QTLs

**Figure 2:**
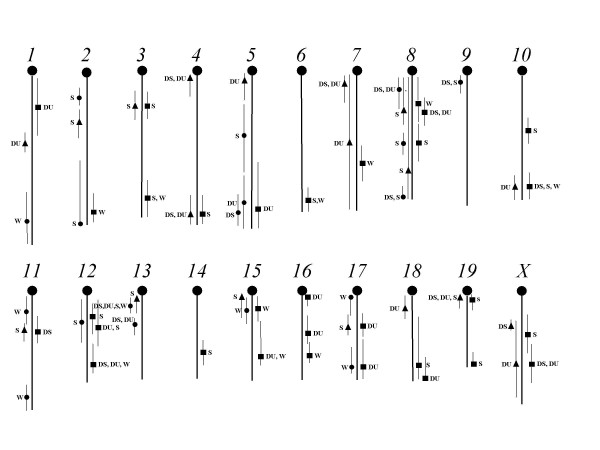
**Locations on each of the chromosomes of direct-effect QTLs for the physical activity/weight traits, relationship QTLs that affect the association of the activity traits and weight, and epistatic QTLs involved in interactions with the relationship QTLs**. Direct-effect QTLs = circles, relationship QTLs = triangles, epistatic QTL = squares, DS = distance, DU = duration, S = speed, and W = body weight.

The results of the scan for direct-effect QTLs affecting body weight are shown in Additional file 2 and are illustrated in Figure 2 (circles). We have designated each QTL as *WT *followed by its chromosome number and an extension to indicate whether the QTL is the first or second on the chromosome. Seven QTLs were discovered in this scan, including two each on chromosomes 11 and 17. Only the QTL on chromosome 13 (*WT13.1*) appears to co-localize with any of the QTLs previously discovered for distance, duration, or speed (Figure 2). Thus the direct-effect QTLs for body weight appear to be generally distinct from those influencing the physical activity traits in this population of mice. Five QTLs are significant at the experiment-wise level whereas two (*WT1.1 *and *WT17.1*) have LPR values that reached chromosome-wise significance. The QTLs contribute individually from 3.3 to 6.3% and collectively 27% (adjusted coefficient of multiple determination from multiple regression) of the total variance of body weight.

Additive genotypic effects are significant for five body weight QTLs, their absolute values averaging 0.33, or about 1/3 of a standard deviation (Additional file 2). The signs of these significant *a *values are mixed, suggesting that for different QTLs, either the C57L/J (positive values) or C3H/HeJ (negative values) alleles increased body weight. Four QTLs also show significant dominance genotypic values, the average of their absolute values of 0.30 being nearly the same as that for the additive effects. The four significant *d *values are also positive in sign, indicating that body weight in the CH heterozygotes is greater than that for the average of the two homozygotes. Two QTLs (*WT11.2 *and *WT17.1*) exhibit overdominance in which the heterozygote is greater than either homozygote.

### Relationship QTLs

A total of 19 *rel*QTLs were discovered affecting one or more of the three activity traits (Additional file 3; Figure 2, triangles). These *rel*QTLs are located on 15 of the 20 chromosomes, including two each on chromosomes 4, 7, 8, and X. Three *rel*QTLs (on chromosomes 8, 13, and 15) map within the confidence interval for the activity traits or for body weight (Figure 2). All LPR scores were significant at the chromosome-wise level, none reaching genome-wise significance. Fifteen of the 19 *rel*QTLs affect the relationship between body weight and one of the three physical activity traits, three (*Act4WT.1*, *Act4WT.2*, *Act7WT.1*) affect two traits (distance and duration), and one *rel*QTL (*Act19WT.1*) significantly affects all three traits. Both *rel*QTLs on chromosome X (*ActXWT.1 *and *ActXWT.2*) are sex-specific, affecting males only; all other *rel*QTLs affect both sexes.

Additional file 4 shows the results of regressions of the physical activity traits on body weight for the three genotypes (HH, CH, and CC) at the SNP marker nearest each of the positions of the 19 *rel*QTLs. As may be seen, there is considerable diversity in the patterns of these regressions among the *rel*QTLs. For the HH, CH, and CC genotypes, respectively, there are 11, 5, and 11 significant regression coefficients, suggesting that homozygotes tend to show a greater association of the physical activity traits with body weight than do heterozygotes. Judging by the signs of the significant regressions, this association often tends to be positive for the CC (6+, 5-) and CH (4+, 1-) genotypes but negative for the HH genotype (4+, 7-). Regression patterns for the four QTLs affecting more than one trait are similar in all cases. Coefficients of determination (*r*^2 ^values) range from 0 to as high as 0.15, and for those associated with significant regressions, average 8%, 5%, and 9%, respectively, for the HH, CH, and CC genotypes.

**Figure 3:**
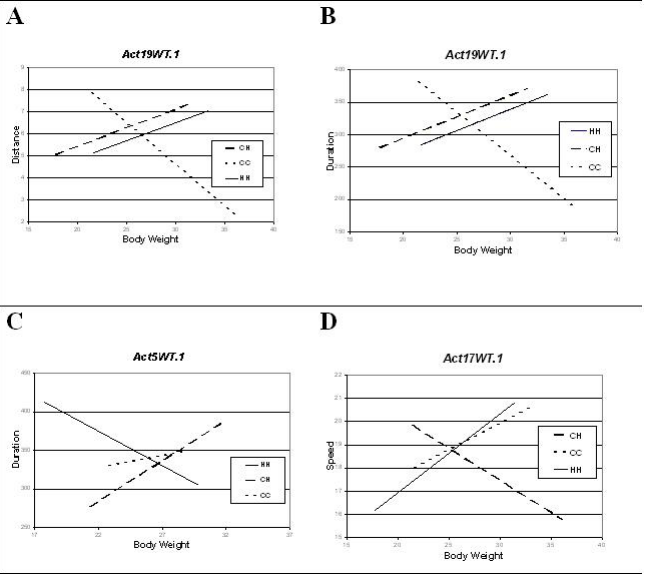
**Examples of the variation in the effects of each of the genotypes at relationship QTLs on the association between physical activity and body weight**. HH = C3H/HeJ homozygotes, CCF = C57/J homozygotes, CH = heterozygotes; plots A and B represent pleiotropic effects of the same relationship QTL on distance and duration; plot C illustrates the effect of a different relationship QTL on duration and plot D illustrates the effect of yet another relationship QTL on speed.

The genotype-dependent nature of the regressions of the activity traits on body weight is illustrated in Figure 3 for three different QTLs. Figures 3A and 3B show that the effect of *Act19WT.1 *on the relationship of both distance and duration with body weight is nearly identical (regression of HH positive, CC negative). However, *Act15WT.1 *(Figure 3C), which also affects the duration/body weight relationship, shows quite a different pattern (regression of CC, CH positive, HH negative). Figure 3D illustrates yet another pattern in which heterozygotes at the *Act17WT.1 *locus show a negative, and homozygotes a positive, association of body weight with speed.

### Epistasis

Additional file 5 gives the results of the genome scan for QTLs showing epistasis with each of the *rel*QTLs. Because of the lack of heterozygosity for loci on the X chromosome in males as well as the reduced sample available for male mice, we tested only the 17 autosomal *rel*QTLs for epistasis, eliminating the two male-specific *rel*QTLs on the X chromosome. This scan uncovered a total of 40 significant interactions involving 31 *epi*QTLs with all autosomal *rel*QTLs except the two on chromosome 7 (*Act7WT.1 *and *Act7WT.2*). The LPR value for one epistatic combination, *Act15WT.1 *with *Act12epi.1*, reached genome-wide significance whereas all others are significant at the chromosome-wise level only. Seven of the *rel*QTLs interact with more than one *epi*QTL, this being noticeable for *Act10WT.1 *(7 *epi*QTLs) and especially for *Act19WT.1 *(9 *epi*QTLs).

The *epi*QTLs are widely dispersed throughout the genome; all chromosomes except 9 and 13 contain at least one *epi*QTL and two chromosomes, 12 and 16, contain three each (Figure 2, squares). Locations of seven of the 31 *epi*QTLs are at or near those for the direct-effect QTLs for weight or the physical activity traits. Also, another nine *epi*QTLs co-localize with *rel*QTLs at identical or very similar positions on these chromosomes (see Figure 2), suggesting that these *epi*QTLs in fact are the same as the *rel*QTLs. Of these nine *epi*QTLs, six exhibit reciprocity in the significant epistatic interactions between QTLs seen on chromosomes 3 and 19, 4 and 11, and 10 and 15 (for example, note the interactions of *Act3WT.1 *with *Act19epi.1*, and *Act19WT.1 *with *Act3epi.1 *in Additional file 5). This provides additional evidence of the commonality of these particular *epi*QTLs with the *rel*QTLs.

With regard to the traits involved in epistasis, seven of the 17 autosomal *rel*QTLs exhibited epistatic interactions with 11 different *epi*QTLs that significantly affected body weight. Although distance and duration are highly correlated, significant epistatic interactions were much more prevalent for duration (five *rel*QTLs with 13 *epi*QTLs) than for distance (three *rel*QTLs with five *epi*QTLs). *Act10WT.1 *had a particularly strong effect on duration through its interactions with six other *epi*QTLs. The number of epistatic interactions affecting speed is similar to that for duration, involving six *rel*QTLs and 10 *epi*QTLs. Clearly, epistatic effects are acting differentially because all 17 *rel*QTLs affecting a specific activity trait (Additional file 4) exert epistatic effects only on that trait or on body weight, not both. This suggests that differential epistasis can account for the variation among the genotype-specific associations of the activity traits and body weight exhibited by the *rel*QTLs (Additional file 4).

**Figure 4:**
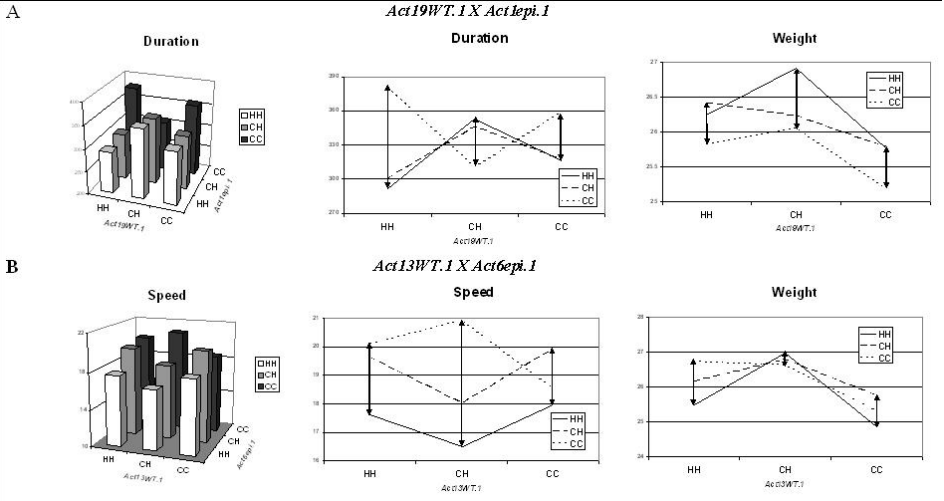
**Two examples of epistatic effects on the association between physical activity and body weight**. Each example includes a bar diagram that shows the epistatic effects of two QTLs on the physical activity trait significantly affected, and two additional line plots that illustrate the effect on the variance (arrows) of both the affected physical activity trait and on body weight from the perspective of the *rel*QTL; note that the physical activity trait is more affected than weight, illustrating differential epistasis.

Two examples of the epistatic interactions of *rel*QTLs and *epi*QTLs are illustrated in Figure 4. Each example includes a bar diagram that shows the epistatic effects of the two QTLs on the physical activity trait significantly affected, and two additional line plots that illustrate the effect on the variance (arrows) of both the affected physical activity trait and on body weight from the perspective of the *rel*QTL. In the first panel in Figure 4A, note the increase in duration from the HH to the CC genotype at the *Act19WT.1 *locus, but only when another epistatic locus on chromosome 1 (*Act1epi.1*) is homozygous, not heterozygous. This epistasis is also seen in the second plot where the lines connecting each of the genotypes are not parallel. The second plot also shows that the variance of duration is greatest for the HH compared to the CH or CC genotypes at the *rel*QTL locus. Body weight was not significantly affected by the interactions of this QTL pair, and this is reflected in the roughly parallel lines in the third plot (Figure 4A) and also the more uniform variances throughout the genotypes. Figure 4B shows that *Act13WT.1 *affects speed and exhibits underdominance when associated with HH or CH genotypes, but overdominance when associated with the CC genotype, at another QTL on chromosome 6. Note again the lack of parallel lines for speed in the second plot but the roughly parallel lines for body weight. In both examples, therefore, epistasis affects the physical activity trait differently from body weight, illustrating differential epistasis.

## Discussion

The purpose of this study was to test for a genetic (pleiotropic) association between the three physical activity traits and body weight in an F_2 _population of mice. To this end, first we mapped body weight QTLs to see whether they might be located near some of the QTLs for the physical activity traits previously mapped [10]. As will be recalled, only one of the seven body weight QTLs (*WT13.1*) co-localized with a QTL affecting the activity traits. Thus at least in this population of mice, it seems clear that the direct-effect QTLs for the physical activity traits are largely independent from those for body weight. However, this conclusion holds only for body weight at the age (average of 12 weeks) the mice were measured and may not be true for weight at other ages. The number of body weight QTLs we discovered was necessarily limited, however, because the inbred progenitors for this particular population were selected on the basis of their divergence in physical activity traits, not body weight. Many more QTLs for body weight measured at various ages have been identified in other populations of mice [26-29]. So we may eventually find that some of these body weight QTLs also exert pleiotropic effects on physical activity traits.

### Indirect QTL effects on physical activity

The search for QTLs that indirectly affected the physical activity traits via their relationship with body weight was quite successful, uncovering 19 different *rel*QTLs spread throughout the genome. At least 15 (79%) of these *rel*QTLs appeared to be distinct from the direct-effect QTLs for the activity traits [10] or for body weight (presented above). This proportion of independent *rel*QTLs is similar to that of 70% (16 of 23) discovered by Cheverud *et al*. [12] for a number of mouse mandibular traits with overall mandible length, but is considerably higher than that of 27% (3 of 11) found by Pavlicev *et al*. [13] affecting the association between limb bone lengths and body weight in an intercross population of mice. Pavlicev *et al*. [13] suggested that since their progenitor strains had been created by selection for large (LG/J) and for small (SM/J) body weights, this may have increased the chance of detecting body weight QTLs that also pleiotropically influenced limb bone lengths. The progenitor strains used to generate our intercross population did not have this history of selection, so perhaps our choice of strains and the traits we measured accounted for the high proportion of independent *rel*QTLs we found. Whatever the case, the 15 *rel*QTLs were concealed in the original scans for direct-effect QTLs because of their opposing effects in mice with large versus small body weights. Their discovery substantially increases the total number of QTLs known to affect the physical activity traits in this population of mice.

Given the initial calculation of the near zero, non-significant phenotypic correlations of body weight with each of the physical activity traits in the total population, it was interesting to see what patterns of genotypic-specific regressions the *rel*QTLs might exhibit. In principle the overall phenotypic independence of body weight from the activity traits could be achieved with some *rel*QTLs showing all positive, and some all negative, regressions (although of different magnitudes for the three genotypes). However, instead, each of the *rel*QTLs had at least one genotype that showed a positive, and one a negative, regression of the activity trait or traits on weight, so body weight showed overall independence at each of these loci. Many (42 of 72) of these regressions actually were not significant, and although this may be partly a consequence of limited statistical power especially for the homozygotes that had lower sample sizes, it is another indication of the general independence of body weight from the activity traits. In contrast, all 75 regressions of limb lengths on body weight calculated by Pavlicev *et al*.[13] for each of three genotypes at the *rel*QTLs they discovered were significant. In addition, the coefficients of determination they calculated averaged 0.23, much higher than that of 0.07 for the significant regressions for the physical activity traits (Additional file 4). Not surprisingly, body weight clearly has a greater association with limb lengths [30,13] than with the physical activity traits we measured in this specific population of mice.

Among the *rel*QTLs, there was no consistent pattern as to which genotype produced a positive, zero, or negative association of the physical activity traits with body weight. There were a few trends previously detailed such as the heterozygotes showing the fewest number of significant regressions, but the effect of a particular genotype at a *rel*QTL on the activity/weight association could not be predicted. However, within those *rel*QTLs that affected the association of body weight with more than one of the physical activity traits, the pattern of genotype-specific regressions was consistent across the traits. As an example, for *Act19WT.1 *the HH and CH genotypes produced positive, and the CC genotype negative, regressions for duration, distance, and speed (Additional file 4). These types of consistent pleiotropic effects produce positive genetic covariances that are compatible with the moderate to high phenotypic correlations among the activity traits. Similar patterns of variability in regressions among *rel*QTLs but consistency within *rel*QTLs were found by Pavlicev *et al*. [13], so may be generally expected in future studies designed to search for *rel*QTLs.

The discovery of the *rel*QTLs in this population of mice is of evolutionary interest because it shows that there is genetic variation in their pleiotropic effects on body weight and the physical activity traits upon which natural selection can act. From the various regression patterns exhibited by the *rel*QTLs, it can be predicted that selection for a particular activity trait such as speed would favor different genotypes with different body weights (Additional file 4, Figure 3). Furthermore, since body weight itself changes, this can result in an increase in the difference among genotypic values of traits affected by these loci, and thus in increases in their variability [13]. Selection favoring genotypes with non-significant (zero) slopes (Additional file 4) could lead to a complete loss of association of body weight with physical activity.

### Differential epistasis

We discovered 40 significant interactions of the *rel*QTLs with 31 separate epistatic QTLs that influenced the physical activity traits and body weight. These numbers are quite comparable to the 40 epistatic interactions involving 33 *epi*QTLs found by Pavlicev *et al*. [13] in their analysis of the association of limb bone lengths with body weight in an entirely different population of mice. In our epistasis scan, *Act19WT.1 *alone accounted for 11 of the 40 significant interactions so it appears to be a particularly important *rel*QTL. It will be recalled that this *rel*QTL was the only one discovered that significantly affected the relationship of body weight with all three physical activity traits (Additional file 3). Another *rel*QTL, *Act10WT.1*, interacted with seven different *epi*QTLs, affecting duration in six of these cases. Therefore, of the 13 epistatic interactions affecting duration, about half involved just this one *rel*QTL. However, with regard to multiple interactions, these two *rel*QTLs were exceptions because all other *rel*QTLs typically interacted with only one or two (or at the most, three) *epi*QTLs.

Each of the interactions significantly affected either a physical activity trait or body weight, but not both, suggesting differential epistasis. In most (28) of the interactions a physical activity trait rather than body weight was affected even though weight was involved in the effects produced by all *rel*QTLs. Wolf *et al*. [25] have also found that the majority of epistatic effects on early- and late-developing skull traits in a population of mice were restricted to single traits, so epistasis may often act in a differential fashion. In any event, differential epistasis appears to satisfactorily account for variation in the genotype-specific associations of the physical activity traits with body weight for each of the *rel*QTLs we discovered. As explained earlier, epistatic interactions involving the *rel*QTLs that produce significant changes for each genotype in the variances of one trait but not the other produce differences in the relationships of these traits as we have documented with regressions.

Although 31 *epi*QTLs were found in the epistasis scans, it is clear that many of them are not unique. As previously detailed, as many as 10 of the *epi*QTLs map near *rel*QTLs and another seven map near direct-effect QTLs for the physical activity traits or for body weight (Figure 2). This suggests that at most 14 of the *epi*QTLs, or less than half of those discovered, appear to be independent from the *rel*QTLs or direct-effect QTLs. It is also possible that some of the epistatic pairs of QTLs we found may be the same as those previously discovered by Leamy *et al*. [11] in their genome scan for epistatic interactions affecting the three physical activity traits in this same population of mice. Therefore, we reviewed those interactions listed as significant at the 0.001 level for each of these traits given in Leamy *et al*. ([11]; Additional files 2, 3, 3) to see whether any matched our results (Additional file 5). None of the 10 interactions for distance or the 12 interactions for duration given by Leamy *et al*.[11] was the same as those we discovered in this study. For speed, however, five of the eight previously found to be significant appear to be the same as five of our interactions, including *epi*QTLs on chromosomes 10, 11 (perhaps the same as *Act11WT.1*), 12, 18 and 19. It is not at all clear why some of the previous interactions found for speed but not distance or duration match those we found, but it emphasizes the difference between our scan that searched for interactions with each of the *rel*QTLs compared to the scan done previously for every two locus combination on each pair of chromosomes.

Clearly, it seems that the QTLs we have uncovered act directly, indirectly, or in both ways on the activity and weight traits. We have classified them into three categories (direct-effect QTLs, *rel*QTLs, and *epi*QTLs) based on the approach we used for their discovery. However, beyond this approach, this distinction may be arbitrary since a direct-effect QTL in one population could well turn out to be a *rel*QTL or an *epi*QTL in another population. All such QTLs collectively contribute to the phenotypic values and variability of the activity and weight traits, suggesting a complex genetic basis for these traits.

### Candidate genes

Although the *rel*QTLs (and *epi*QTLs) we have found provide approximate locations throughout the genome for genes that affect the physical activity traits via their association with body weight, the identity of these genes is presently entirely unknown. Hundreds of potential candidate genes lie within the confidence intervals of many of these QTLs, so it would seem presumptuous to attempt to list possible candidates for them. Some consideration of potential candidate genes seems warranted, however, for one specific *rel*QTL: *Act19WT.1. Act19WT.1 *exhibited the highest LPR value that in fact nearly reached genome-wise significance, and in addition, this *rel*QTL showed the greatest number of significant epistatic interactions (recall Additional file 5 results). Therefore, we searched the Mouse Genome Informatics database [31] for potential candidate genes in the area of this *rel*QTL. However, the possibilities listed below are only meant to be illustrative and in no way are exhaustive.

For *Act19WT.1*, one potential candidate gene is *IGHMBP*, immunoglobulin mu binding protein-2 (chromosome 19, 0 cM). This gene affects the cardiovascular and muscle systems as well as growth, and is apparently essential for cardiomyocyte maintenance necessary to meet respiratory demands [32]. Another possibility is *SCYL1*, Scy1-like 1(chromosome 19, 6 cM), that affects muscle tone, behavior, growth/size, and the nervous system [33]. A third and perhaps most interesting potential candidate gene is *ACTN3*, actinin alpha 3 (chromosome 19, 3 cM). In humans, a nonsense polymorphism at this locus is quite common and is associated with reduced muscle strength and sprint performance [34,35]. In mice, knockouts exhibit reduced force generation, apparently because of a shift from the properties of fast muscle fibers to those of slow muscle fibers [36]. Interestingly, an isoform of *ACTN3*, actinin alpha 2 (*Actn2 *on chromosome 13, 7 cM) that has similar physiological functioning as *ACTN3*, is located near the significant single-effect QTLs for the physical activity traits (*DIST13.1*, 11 cM; *DUR13.1*, 11 cM; *SPD13.1*, 9 cM) we discovered earlier [10].

These few examples provide some insight into the range of genes that might affect the relationship between physical activity and body weight. They also illustrate the complexity of this relationship and how difficult it is to even know which systems (nervous, muscular, cardiovascular, endocrine, etc.) may be involved. However, some recent studies by Good and colleagues [37-39] provide clear evidence of one example in which the nervous and endocrine systems are involved in the linkage between body weight with physical activity. Good *et al*. [37] have shown that *NHLH2*, nescient helix loop helix 2, is expressed in neuroendocrine tissues such as the pituitary and hypothalamus and acts to reduce physical activity in mice that eventually leads to adult-onset obesity. *NHLH2 *may exert its effects by regulating the motivation for voluntary physical activity, but whatever the actual pathway, this gene clearly produces a negative association between activity and body weight. *NHLH2 *is located on chromosome 3, although not in the area of the *rel*QTL (*Act3WT.1*) that we discovered on this chromosome.

## Conclusion

We discovered a number of *rel*QTLs in our population of mice that provided evidence for a genetic association of physical activity and body weight. Genotypes at these loci exhibited variously positive, zero, and negative activity/weight associations, and their individual and collective net effect produced overall independence of body weight from physical activity. However, even where plots of physical activity versus body weight show no association, some of the variability we typically see in such plots may be due to unique combinations of genotypes carried by individuals at their *rel*QTLs. Since we have seen that the *rel*QTLs appear to be generated from differential epistatic effects, it may prove very difficult to predict the level of physical activity an individual with a specific body weight might voluntarily achieve. Our discovery of *rel*QTLs in this population of mice also suggests that the genetic architecture of physical activity and its relationship to body weight may turn out to be even more complex than we had imagined.

## Competing interests

The authors declare that they have no competing interests.

## Authors' contributions

LJL performed the data analysis, wrote and prepared the manuscript for submission. JTL was the principal supervisor of the study and assisted with preparation of the manuscript. DP reviewed the manuscript and all authors read and approved the final manuscript.

## Supplementary Material

Additional file 1 **Basic statistics for body weight and physical activity traits.** Shown are the means and standard deviations for distance, duration, and speed in the 310 F_2 _mice, and pairwise correlations among these four traits. * = P < 0.05; ** = P < 0.01 Click here for file

Additional file 2 **QTLs for body weight**. Shown are the locations, confidence intervals (CI), LPR scores (log_10_Prob^-1^), percentage of the variation explained (%), and standardized additive (*a*) and dominance genotypic values (*d*) for QTLs on all chromosomes (Ch) significantly affecting body weight. Locations are given as map distances from the nearest proximal marker (Marker Dist) and from the centromere (Cent. Dist), and confidence intervals are expressed from the centromere; all LPR values are significant at the 5% chromosome-wise level and those exceeding 3.80 are significant at the 5% experiment-wise level. * = P < 0.05; ** = P < 0.01. Click here for file

Additional file 3 **Relationship QTL (*rel*QTL) significantly affecting the association of the physical activity traits (distance, duration, or speed) with body weight**. Locations of these *rel*QTL on each chromosome (Chr) are shown in terms of the distance in cM proximal (-) or distal (+) to the nearest SNP marker and from the centromere; support intervals around the locations are expressed as cM from the centromere; LPR (log of the probability) values are derived from single trait analyses, or where more than one trait is pleiotropically affected, from multiple trait analyses; *rel*QTLs on chromosome X affect males (denoted by *M *subscripts) only. Click here for file

Additional file 4 **Regressions (*b*) of the physical activity traits on body weight for C3H/HeJ homozygotes (HH), C57L/J homozygotes (CC) and heterozygotes (CH) at each of the *rel*QTLs**. *r*^2 ^= coefficients of determination; * = P < 0.05; ** = P < 0.01 Click here for file

Additional file 5 **Epistatic QTLs (*epi*QTLs) that significantly interact with the *rel*QTLs to affect the physical activity traits (distance, duration, or speed) or body weight**. Locations of these *epi*QTL on each chromosome (Chr) are shown in terms of the distance in cM proximal (-) or distal (+) to the nearest SNP marker and from the centromere; support intervals around the locations are expressed as cM from the centromere; LPR = log of the probability Click here for file
