# Supplementary material for: Genetic variation in the pleiotropic association between physical activity and body weight in mice
Source: Genet Sel Evol. 2009 Sep 23;41(1):41. doi: 10.1186/1297-9686-41-41 (PMC2760520; doi:10.1186/1297-9686-41-41)
Supplement: Additional file 1 — Basic statistics for body weight and physical activity traits. Shown are the means and standard deviations for distance, duration, and speed in the 310 F2 mice, and pairwise correlations among these four traits. * = P < 0.05; ** = P < 0.01 [file 1297-9686-41-41-S1.pdf]

Table 1 - Basic statistics for body weight and physical activity traits

			Correlations		
	Mean	Std Dev	Distance	Duration	Speed
Weight (g)	26.23	2.30	0.04	0.05	0.04
Distance (km/day)	6.35	2.38		0.92**	0.71**
Duration (min/day)	330.29	100.83			0.45**
Speed (meters/min)	18.68	3.41			

Shown are the means and standard deviations for distance, duration, and speed in the 310 F_2 mice, and pairwise correlations among these four traits.
* = P < 0.05; ** = P < 0.01
