# Supplementary material for: Genetic variation in the pleiotropic association between physical activity and body weight in mice
Source: Genet Sel Evol. 2009 Sep 23;41(1):41. doi: 10.1186/1297-9686-41-41 (PMC2760520; doi:10.1186/1297-9686-41-41)
Supplement: Additional file 2 — QTLs for body weight. Shown are the locations, confidence intervals (CI), LPR scores (log10Prob-1), percentage of the variation explained (%), and standardized additive (a) and dominance genotypic values (d) for QTLs on all chromosomes (Ch) significantly affecting body weight. Locations are given as map distances from the nearest proximal marker (Marker Dist) and from the centromere (Cent. Dist), and confidence intervals are expressed from the centromere; all LPR values are significant at the 5% chromosome-wise level and those exceeding 3.80 are significant at the 5% experiment-wise level. * = P < 0.05; ** = P < 0.01. [file 1297-9686-41-41-S2.pdf]

Table 2 - QTLs for body weight

QTL	Ch	Marker	Marker Dist	Cent. Dist	CI	LPR	%	*a/SD*	*d/SD*
WT1.1	1	*rs3699304*	+4	97	67—115	2.80	4.82	-0.26**	0.27*
WT11.1	11	*mCV24434350*	+10	14	4—22	3.88	6.37	-0.32**	0.27*
WT11.2	11	*rs6370458*	+4	88	74—98	2.00	3.29	-0.01	0.37**
WT13.1	13	*rs6329684*	+10	11	3—23	2.97	5.44	0.37**	-0.15
WT15.1	15	*rs13482429*	+14	18	8—36	4.02	6.22	-0.37**	0.10
WT17.1	17	*rs6309949*	0	5	5—29	1.82	2.88	0.11	0.29*
WT17.2	17	*rs3660112*	0	51	41—57	3.23	5.01	0.31**	-0.14

Shown are the locations, confidence intervals (CI), LPR scores ($\log_{10}\text{Prob}^{-1}$), percentage of the variation explained (%), and standardized additive (*a*) and dominance genotypic values (*d*) for QTLs on all chromosomes (Ch) significantly affecting body weight. Locations are given as map distances from the nearest proximal marker (Marker Dist) and from the centromere (Cent. Dist), and confidence intervals are expressed from the centromere; all LPR values are significant at the 5% chromosome-wise level and those exceeding 3.80 are significant at the 5% experiment-wise level. * = $P < 0.05$; ** = $P < 0.01$.
