# Supplementary material for: Genetic variation in the pleiotropic association between physical activity and body weight in mice
Source: Genet Sel Evol. 2009 Sep 23;41(1):41. doi: 10.1186/1297-9686-41-41 (PMC2760520; doi:10.1186/1297-9686-41-41)
Supplement: Additional file 3 — Relationship QTL (relQTL) significantly affecting the association of the physical activity traits (distance, duration, or speed) with body weight. Locations of these relQTL on each chromosome (Chr) are shown in terms of the distance in cM proximal (-) or distal (+) to the nearest SNP marker and from the centromere; support intervals around the locations are expressed as cM from the centromere; LPR (log of the probability) values are derived from single trait analyses, or where more than one trait is pleiotropically affected, from multiple trait analyses; relQTLs on chromosome X affect males (denoted by M subscripts) only. [file 1297-9686-41-41-S3.pdf]

Table 3 - Relationship QTL (*rel*QTL) significantly affecting the association of the physical activity traits (distance, duration, or speed) with body weight

Chr	*rel*QTL Name	Nearest SNP Marker	Marker distance	Centromere distance	Support interval	LPR	Traits
1	*Act1WT.1*	*rs6293581*	+4	38	26—46	2.35	Duration
2	*Act2WT.1*	*rs4223211*	0	40	30—50	2.50	Speed
3	*Act3WT.1*	*rs6212539*	-2	30	22—40	2.84	Speed
4	*Act4WT.1*	*rs13477568*	0	8	8—24	2.28	Distance, Duration
4	*Act4WT.2*	*gnf04.133.236*	1	91	76—98	2.36	Distance, Duration
5	*Act5WT.1*	*CEL-5_11863229*	+2	5	3—15	2.26	Duration
7	*Act7WT.1*	*rs13479600*	+8	10	2—18	2.43	Distance, Duration
7	*Act7WT.2*	*rs13479412*	-6	46	2—83	2.43	Duration
8	*Act8WT.1*	*rs3659852*	-4	28	2—42	2.63	Speed
8	*Act8WT.2*	*rs13479996*	-4	63	0—84	2.63	Speed
10	*Act10WT.1*	*rs13480786*	+4	78	64—84	2.16	Duration
11	*Act11WT.1*	*rs6205294*	+8	27	19—43	2.36	Speed
13	*Act13WT.1*	*rs6329684*	0	1	1—17	1.99	Speed
15	*Act15WT.1*	*rs13482429*	+4	8	4—18	2.10	Speed
17	*Act17WT.1*	*rs13483021*	+2	38	30—48	2.12	Speed
18	*Act18WT.1*	*rs3670421*	+8	12	4—30	2.14	Duration
19	*Act19WT.1*	*rs3671328*	+2	7	5—21	3.64	Distance, Duration, Speed
X	$ActXWT.1_M$	*rs13483781*	0	33	31—35	2.66	Distance
X	$ActXWT.2_M$	*rs13483890*	+4	54	32—82	2.60	Duration

Locations of these *rel*QTL on each chromosome (Chr) are shown in terms of the distance in cM proximal (-) or distal (+) to the nearest SNP marker and from the centromere; support intervals around the locations are expressed as cM from the centromere; LPR (log of the probability) values are derived from single trait analyses, or where more than one trait is pleiotropically affected, from multiple trait analyses; *rel*QTLs on chromosome X affect males (denoted by *M* subscripts) only.
