# Supplementary material for: Genetic variation in the pleiotropic association between physical activity and body weight in mice
Source: Genet Sel Evol. 2009 Sep 23;41(1):41. doi: 10.1186/1297-9686-41-41 (PMC2760520; doi:10.1186/1297-9686-41-41)
Supplement: Additional file 4 — Regressions (b) of the physical activity traits on body weight for C3H/HeJ homozygotes (HH), C57L/J homozygotes (CC) and heterozygotes (CH) at each of the relQTLs. r2 = coefficients of determination; * = P < 0.05; ** = P < 0.01 [file 1297-9686-41-41-S4.pdf]

Table 4 - Regressions (*b*) of the physical activity traits on body weight for C3H/HeJ homozygotes (HH), C57L/J homozygotes (CC) and heterozygotes (CH) at each of the *rel*QTLs

		HH		CH		CC	
QTL	Trait	*b*	r^2	*b*	r^2	*b*	r^2
Act1WT.1	Duration	12.59*	0.08	3.48	0.01	-9.82	0.05
Act2WT.1	Speed	0.29	0.04	0.25	0.02	-0.38**	0.09
Act3WT.1	Speed	0.43*	0.08	-0.27	0.03	0.23	0.02
Act4WT.1	Distance	-0.33*	0.07	0.04	0.00	0.27*	0.06
	Duration	-14.61*	0.08	0.56	0.00	14.69**	0.09
Act4WT.2	Distance	-0.00	0.00	-0.11	0.01	0.36**	0.11
	Duration	-3.13	0.00	-4.29	0.01	16.33**	0.13
Act5WT.1	Duration	-8.89	0.05	10.55**	0.05	3.15	0.01
Act7WT.1	Distance	0.28**	0.10	-0.12	0.01	0.07	0.00
	Duration	11.19**	0.08	-5.11	0.01	3.59	0.01
Act7WT.2	Duration	-1.16	0.00	7.77	0.02	-5.26	0.02
Act8WT.1	Speed	0.44*	0.09	-0.17	0.01	0.18	0.0
Act8WT.2	Speed	-0.37*	0.08	0.05	0.00	0.19	0.02
Act10WT.1	Duration	-9.26	0.02	2.17	0.00	14.72**	0.10
Act11WT.1	Speed	-0.02	0.00	0.37**	0.05	-0.21	0.01
Act13WT.1	Speed	-0.31*	0.05	0.03	0.00	0.50**	0.10
Act15WT.1	Speed	-0.43**	0.10	0.20	0.02	-0.06	0.00
Act17WT.1	Speed	0.34	0.04	-0.28*	0.03	0.23	0.02
Act18WT.1	Duration	7.51	0.02	3.68	0.01	-11.45**	0.09
Act19WT.1	Distance	0.16	0.03	0.17*	0.03	-0.38**	0.09
	Duration	6.56	0.02	6.59*	0.03	-13.30*	0.06
	Speed	0.38	0.05	0.20	0.02	-0.61**	0.15
$ActXWT.1_M$	Distance	-0.23	0.06			0.11	0.01
$ActXWT.2_M$	Duration	-11.71*	0.09			6.26	0.02

r^2 = coefficients of determination; * = P < 0.05; ** = P < 0.01
