# Supplementary material for: Genetic variation in the pleiotropic association between physical activity and body weight in mice
Source: Genet Sel Evol. 2009 Sep 23;41(1):41. doi: 10.1186/1297-9686-41-41 (PMC2760520; doi:10.1186/1297-9686-41-41)
Supplement: Additional file 5 — Epistatic QTLs (epiQTLs) that significantly interact with the relQTLs to affect the physical activity traits (distance, duration, or speed) or body weight. Locations of these epiQTL on each chromosome (Chr) are shown in terms of the distance in cM proximal (-) or distal (+) to the nearest SNP marker and from the centromere; support intervals around the locations are expressed as cM from the centromere; LPR = log of the probability [file 1297-9686-41-41-S5.pdf]

Table 5 - Epistatic QTLs (*epi*QTLs) that significantly interact with the *rel*QTLs to affect the physical activity traits (distance, duration, or speed) or body weight

*rel*QTL	*epi*QTL	Chr	Nearest SNP marker	Marker distance	Centr. distance	Support interval	LPR	Traits
Act1WT1	*Act18epi.2*	18	*rs13483484*	0	64	46—64	2.04	Duration
Act2WT.1	*Act3epi.2*	3	*rs6256060*	+4	84	52—98	2.32	Weight
	Act6epi.1	6	*rs13479096*	-2	93	88—96	2.29	Weight
Act3WT.1	*Act19epi.1*	19	*rs3671328*	+4	9	5—23	2.43	Speed
Act4WT.1	*Act8epi.2*	8	*rs3659852*	0	32	18—46	2.53	Distance
	Act8epi.2	8	*rs3659852*	0	32	18—46	2.70	Duration
Act4WT.2	*Act11epi.1*	11	*rs3023265*	-4	30	20—40	2.48	Distance
Act5WT.1	*Act7epi.1*	7	*rs13479412*	+8	60	44—72	2.50	Weight
	Act16epi.3	16	*rs4199410*	+4	51	39—59	2.55	Weight
	Act17epi.1	17	*rs13483021*	-2	34	26—46	2.72	Duration
Act7WT.1	*Act16epi.1*	16	*rs4155963*	0	1	1—5	2.28	Duration
Act7WT.2	*Act8epi.3*	8	*rs3705695*	-2	50	42—62	2.37	Weight
Act10WT.1	*Act2epi.1*	2	*rs3664044*	-4	103	95—107	2.78	Duration
	Act5epi.1	5	*rs13478553*	+4	99	69—109	2.33	Duration
	Act12epi.2	12	*CEL_12_332562*	-2	33	7—29	2.22	Duration
	Act15epi.1	15	*CEL_15_523577*	-6	14	4—30	2.80	Weight
	Act15epi.2	15	*gnf15.090.425*	0	53	37—65	2.19	Duration
	Act16epi.2	16	*rs4175617*	0	31	19—39	2.51	Duration
	Act17epi.2	17	*rs3660112*	0	51	37-55	2.76	Duration
Act11WT.1	*Act4.epi.1*	4	*gtnf04.133.236*	+2	92	74—100	2.89	Speed
	Act8epi.3	8	*rs3023193*	+2	49	41—83	2.23	Weight
	Act15epi.2	15	*gnf15.090.425*	-4	49	29—61	2.21	Weight
Act13WT.1	*Act6epi.1*	6	*rs13479096*	0	95	89—95	2.22	Speed
	Act18epi.1	18	*rs13483417*	+8	55	27—65	2.66	Speed
Act15WT.1	*Act10epi.2*	10	*rs3676330*	-2	82	72.86	2.30	Weight
	Act12epi.1	12	*CEL-12_3325623*	+4	25	13—31	3.90	Speed
	Act19epi.2	19	*rs13483689*	0	55	49—55	2.99	Speed
Act17WT.1	*Act3epi.2*	3	*rs6256060*	+6	86	72—96	2.39	Weight
Act18WT.1	*Act12epi.3*	12	*rs6361467*	-8	60	42—68	2.40	Weight
Act19WT.1	*Act1epi.1*	1	*rs13475816*	0	17	3—31	2.15	Duration
	Act3epi.1	3	*rs6212539*	-2	30	24—38	2.40	Speed
	Act8epi.1	8	*CEL_8_2567770*	+6	22	0—42	2.38	Weight
	Act10epi.1	10	*rs13480660*	-4	42	32—54	3.16	Speed
	Act10epi.2	10	*rs13480786*	0	74	60—80	2.36	Distance
	Act12epi.3	12	*rs6361467*	-8	60	48—68	2.93	Distance
	Act12epi.3	12	*rs6361467*	-6	62	36—68	2.18	Duration
	Act14epi.1	14	*rs3708665*	+8	54	40—62	2.17	Speed
	ActXepi.1	X	*rs13483890*	0	39	23—67	2.13	Speed
	ActXepi.2	X	*CEL-X 10366575*	+4	54	40—74	2.36	Distance
	ActXepi.2	X	*CEL-X 10366575*	+4	54	42—74	2.29	Duration

Locations of these *epi*QTL on each chromosome (Chr) are shown in terms of the distance in cM proximal (-) or distal (+) to the nearest SNP marker and from the centromere; support intervals around the locations are expressed as cM from the centromere; LPR = log of the probability
